# Barriers and Facilitators to Program Sustainability Among State Tobacco Control Programs

**DOI:** 10.5888/pcd21.230211

**Published:** 2024-02-01

**Authors:** Karin Han, Jessica Gannon, Sarah Moreland-Russell

**Affiliations:** 1 Prevention Research Center, Brown School, Washington University in St. Louis, St. Louis, Missouri

## Abstract

Public health programs, particularly tobacco control programs (TCPs) in state health departments, face numerous barriers and facilitators to sustainability, which affect delivery and, consequently, health outcomes achieved. We used the Program Sustainability Framework to review and analyze qualitative interview data from states that received training and technical assistance during the Plans, Actions, and Capacity to Sustain Tobacco Control (PACT) study to better understand the barriers and facilitators to sustainability capacity that these public health programs face at the state level. The PACT study was a multiyear, randomized controlled trial to assess the effectiveness of an action planning workshop and technical assistance in improving capacity for sustainability among 11 intervention and 12 control TCPs. Technical assistance calls focused on the progress and barriers of implementing the sustainability action plan created during the in-person workshops. Calls were audio recorded and professionally transcribed. Thematic analysis focused on the codes describing barriers and facilitators faced by TCPs in increasing their capacity for sustainability. Barriers were reported in the Organization Capacity, Environmental Support, Partnerships, Communication, and Funding Stability domains of the Program Sustainability Framework. Facilitators to action planning and building capacity for program sustainability were primarily in the Strategic Planning, Program Evaluation, Program Adaptation, and Partnership domains. Our study is the first to identify barriers and facilitators to increasing the capacity of program sustainability in TCPs. This work advances the understanding of program sustainability capacity and technical assistance for public health programs.

**Summary**

**What is already known on this topic?**

Public health programs, particularly tobacco control programs in state health departments, face numerous barriers and facilitators to sustainability, which affect the delivery and, consequently, the health outcomes achieved.

**What is added by this report?**

This study is the first to identify barriers and facilitators to increasing the capacity of program sustainability in tobacco control programs.

**What are the implications for public health practice?**

This work advances the understanding of program sustainability capacity and technical assistance for public health programs. Through addressing these barriers and cultivating these facilitators, state health departments can build stronger public health programs.

## Introduction

Programs are able to continuously deliver benefits only if they can sustain their activities over time (1). Sustainability capacity is broadly defined as the existence of structures and processes that allow a program to leverage resources to effectively implement and maintain evidence-based policies and activities (2). Public health programs face numerous barriers and facilitators to sustainability, which affect delivery and, consequently, the health outcomes achieved. Program sustainability is an important factor to evaluate and improve on for successful public health program practice. Tobacco control programs (TCPs) in state health departments face many barriers in achieving program sustainability, such as low and fluctuating funding levels (3), scarce levels of legislative support (4), and lack of organizational capacity (5).

One method to assess and understand program sustainability capacity is use of the Program Sustainability Framework (2). This framework structures programmatic factors into 8 sustainability domains: Environmental Support (having a supportive internal and external climate), Funding Stability (establishing a consistent financial base), Partnerships (cultivating connections between a program and its working partner), Organizational Capacity (having the internal support and resources needed to effectively manage a program), Program Evaluation (assessing a program to inform planning and document results), Program Adaptation (taking actions that adapt a program to ensure its ongoing effectiveness), Communications (strategic engagement with partners and the public), and Strategic Planning (using processes that guide a program’s directions, goals, and strategies) (2).

The framework can be used to develop program goals and assess growth over time toward achieving those goals (6). It has been used to understand capacity for sustainability across many different types of programs (eg, local and state-level health departments, nonprofit organizations) and among high-performing and low-performing programs, providing a better understanding of factors that affect performance and success (6).

## Purpose and Objectives

This evaluation used the Program Sustainability Framework to review and analyze qualitative interview data from states that received training and technical assistance during the Plans, Actions, and Capacity to Sustain Tobacco Control (PACT) study (7) to better understand barriers and facilitators to sustainability capacity faced by these public health programs at the state level. Understanding these factors for TCPs will help programs in addressing barriers, cultivating facilitators, and providing technical assistance to achieve sustainability and ultimately achieve better health outcomes.

## Intervention Approach

The PACT study team developed the Program Sustainability Action Planning Model and Training Curricula and tested its effectiveness in increasing capacity for sustainability among state-level TCPs, as defined by organizational outcomes and Program Sustainability Assessment Tool (PSAT) scores (1) through a multiyear, randomized controlled trial. At the beginning of the study, programs were randomized into control or intervention groups by stratifying the 50 US states into quadrants on the basis of need and tobacco control policy environment. Three programs with varying degrees of funding were selected from each quadrant and pair matched with programs on the basis of the same 3 characteristics. Each pair was then randomized into control (n = 12) or intervention (n = 12). One of the intervention programs dropped out of the study, leaving 11 programs in the intervention group. The intervention states received the training curricula through tailored, action-oriented in-person workshops at baseline. During these workshops, state level TCP staff and other concerned groups participated in learning about sustainability and strategies for developing an action plan. Participants then outlined 1 or 2 specific, measurable, achievable, relevant, time-bound (SMART) objectives (8) for 1 of the 8 Program Sustainability Framework domains and developed an action plan. These action plans included time-specific activities to be shared across the group and tracked over time (7). After the workshops took place, intervention states received ongoing, robust technical assistance throughout their 2-year participation period within the 2018–2022 time frame. Technical assistance included regularly scheduled calls to review action plan progress and access to sustainability resources.

## Evaluation Methods

Technical assistance focused on progress and barriers to implementing the sustainability action plan created during the in-person workshops and consisted of 30- to 45-minute telephone or Zoom calls with the state’s TCP program manager. When program managers were unavailable, other qualified individuals recommended by the program manager substituted.

The telephone and Zoom calls were audio recorded and professionally transcribed (rev.com) for coding in NVivo version 20 (QSR International). Three researchers (including J.G. and S.M.R) coded transcripts until they reached substantial interrater reliability (κ = 0.72). For this study, inductive thematic analysis focused on codes for barriers and facilitators faced by TCPs in increasing their capacity for sustainability. We used the Program Sustainability Framework to structure the themes. The institutional review board at Washington University in St. Louis approved this study.

Eleven states participated in technical assistance interviews during their 2-year study participation. A total of 46 telephone and Zoom calls occurred between January 2019 and January 2022, with each state receiving an average of 2 calls per year.

## Results

### Barriers

Barriers were reported in the Organization Capacity, Environmental Support, Partnerships, Communication, and Funding Stability domains (Table 1). No barriers were reported in the Program Evaluation, Program Adaptation, or Strategic Planning domains.

**Table 1:** Barriers to Increasing the Sustainability Capacity of Tobacco Control Programs: Results of Technical Assistance Interviews (n = 46) in 11 States, January 2019–January 2022

Theme	Direct quote	CDC-defined region	Domains chosen by state tobacco control program^a^
**Organizational Capacity**			
Staffing problems	So, we had years’ worth of work to put together when we once had what five or six staff and then it was just down to just [name] and myself. We were the ones responsible for covering prevention and secondhand smoke cessation and any of that kind of thing.	South	Partnerships
	We actually did the interviews, found somebody that we were going to hire, then before we could actually do the hiring, our reorg plan ended up not being approved, which affected that position. . . . In the meantime, our hiring for that partnership position’s been put on hold until that new plan is approved by DOH [department of health] administration.	South	Partnerships, Communications
	We’ve been trying to fill this position . . . for several months now and have just had what seems like a series of challenges. From a restructuring related to this position that we had proposed and thought it was going to be approved and then ended up not being approved, to this is our second round of interviews now.	South	Partnerships, Communications
	Honestly, I think our key challenge is just lack of person power or even lack of the ability of the people who own certain portions to make it their absolute priority to get it done.	West	Strategic Planning
	But when you constantly keep having vacancies . . . you gain a person, but then you lose another person, and then the process, in and of itself, becomes just daunting.	Midwest	Organizational Capacity, Partnerships
Competing priorities with vaping and e-cigarettes	I think there is definitely a need for more prevention around vaping. The schools really have been requesting a lot, whether it’s educational materials, presentations. But we’re all at capacity and then some.	West	Communications, Funding Stability
	E-cigarettes are a problem and that’s where our focus is right now, with legislation.	West	Communications, Funding Stability
	Plus, we had that emergency ban on vapor products, flavored vapor products that kind of inundated our time.	Midwest	Organizational Capacity, Partnerships
Competing priorities or reassignment to COVID-19	Definitely we’re stretched a little bit thin, for example, I’m on the COVID communications task force.	West	Strategic Planning
	So, I think that it has been most detrimental to our program, the vacancies in COVID. I think COVID has really impacted our contractors and grantees locally, their ability to be able to perform for us, not us performing for anybody else.	Midwest	Organizational Capacity, Partnerships
	And we also were impacted with COVID and capacity, so we definitely had a plan, and we had a product to share with legislators, but one of our staff has been and remains full-time on COVID.	Northeast	Program Evaluation, Communications
**Environmental Support**			
Legislative support	It seemed like we were still, this is an extremely busy tobacco session for us. . . . The things that our partners were needing were not necessarily things that we anticipated that just was based on what showed up in the legislation and some of the things that showed up just weren’t things that we had anticipated showing up.	South	Partnerships, Communications
	How do we want to parse out the meager resources we have in that area? We are very short on lobbyists in this state that are pro-health, pro tobacco control.	South	Partnerships
	This isn’t a bill that we shepherded all the way through the very end of session last year. So, it’s not as though this is the first time we’re trying to work on this initiative. But at the end of the day, you can do all the right things, and it doesn’t mean the legislatures are going to do what you want them to do.	West	Communications, Funding Stability
	So, we had an election in November and in [state] we went from purple to red all the way down from top to bottom and so that’s limiting messaging from us or to partners from us and things like that because we know that we’re trying to be in a defensive posture right now rather than offensive.	Northeast	Program Evaluation, Communications
**Partnerships**			
Formalized commitments	I think where we have really fallen short is not having written agreements with these partners. And that’s been a challenge, I think, for both us at the department side and the partnering agencies, because it’s become such a legal issue to have a memorandum of understanding or a memorandum of agreement.	South	Partnerships, Communications
Partner engagement	It’s just more of a challenge now, too, with COVID because I know everybody has their own priorities, to keep those new partners engaged and active, so we’re still working to do that. And somehow even in this COVID environment, revitalize the efforts of the tobacco control network and get people energized and interested in participating again.	South	Partnerships, Communications
	Trying to make sure that partners were continually engaged and that when they left the meeting, that they felt empowered enough to actually follow through on activities that have been discussed. Because sometimes we would have meetings, and everybody would leave with what they were supposed to do but when we met again there hadn’t been any movement on anything.	South	Partnerships, Communications
	It’s more like one of the frustrations that I always had was getting the people, the stakeholders in our work group engaged, getting them to reply to emails, getting them to take the survey again, it being different people.	West	Partnerships, Communications
Partner representation	We saw perhaps some gaps in some members that were not at the table. And some of the members talked about an outreach to those who we felt were not there. And then, I think after that, we also saw that there were too many representatives from certain agencies that were filling the room that could make room for other agencies. If that makes sense.	Northeast	Communications
**Communications**			
Communication with partners	We did the things we plan to do with the communications plan, but I don’t know that we’ve met our overall goal of trying to be proactive in providing that information to our partners.	South	Partnerships, Communications
Communication methods	So, communications as a whole has been challenging, just because there are different issues with who we can contract with for maybe media or health communications work.	Northeast	Communications
COVID-19–related communications	We had no communications plan, and then COVID came along and we weren’t allowed to put out any communications that didn’t have to do with COVID.	Midwest	Communications
	Our media agency, we volunteered their time to create COVID messaging for the department because of obviously the pandemic and the need to get people vaccinated and tested. It was easier to use the existing contract than to try to contract with someone else. . . . And so it took them away from working with us for quite some time . . . for several months.	West	Partnerships, Communications
**Funding Stability**			
Fluctuations in funding	But when you ask about stability, we’re going to be nervous this coming session with the state looking like a deficit, figuring out where they want to take some money from. And so, it’s a major issue to always protect this. . . . It’s there, but it could be totally taken away.	West	Communications, Funding Stability
	We got from a disputed master settlement fund agreement about 10 to 12 million dollars a year in addition to our federal funds, but that money has now run out in the middle of COVID. We’re really going to be fighting to get that money out of our general revenue fund dedicated to our program. And so, we’ve had to do a lot of work and justifying and creating documents and working on the budget.	Midwest	Communications

Abbreviation: CDC, Centers for Disease Control and Prevention.

a The Program Sustainability Framework structures programmatic factors into 8 sustainability domains: Environmental Support, Funding Stability, Partnerships, Organizational Capacity, Program Evaluation, Program Adaptation, Communications, and Strategic Planning (2). Each tobacco control program chose to address 1 or 2 domains as part of an action plan.

**Organizational Capacity**. In this domain, the main barrier for action plan implementation and capacity building was staffing problems. Interviewees reflected on how lack of personnel within their department affected their ability to execute their sustainability action plan. Another barrier to organizational capacity was competing priorities with vaping and e-cigarette prevention efforts. Additionally, interviewees noted their programs had decreased capacity because of the focus on the COVID-19 pandemic.

**Environmental Support.** In this domain, the main barrier for action plan implementation and capacity building was low or uncertain levels of legislative support from state legislatures. This lack of support concerned resource allocation, funding, and legislation that supports TCPs.

**Partnerships.** In this domain, the main barrier for action plan implementation and capacity building was lack of formalized commitment between TCPs and their partners. Another barrier was lack of engagement from partners, which created difficulties in achieving progress with initiatives, as partners prioritized other work or were unwilling to contribute to shared work. Finally, programs found that some partners — such as the general public, state boards, and professional organizations — were not represented during their in-person workshop. The lack of these additional perspectives prevented holistic partner-centric work from moving forward.

**Communications.** In this domain, limitations to message content and message type were barriers to increasing capacity. Needing to reassign staff to COVID-19–related communications prevented TCP communications for some states. Finally, lack of strong partner communication was thought to impede sustainability capacity in this domain.

**Funding Stability.** In this domain, fluctuating and uncertain funding levels concerned states. Uncertain funding forced program managers to prioritize advocating for more funding over doing other program work and made it difficult to plan for upcoming program goals.

### Facilitators

Facilitators to action planning and building capacity for program sustainability primarily were in the domains of Strategic Planning, Program Evaluation, Program Adaptation, and Partnerships (Table 2).

**Table 2:** Facilitators for Increasing the Sustainability Capacity of Tobacco Control Programs: Results of Technical Assistance Interviews (n = 46) in 11 States, January 2019–January 2022

Theme	Direct quote	CDC-defined region	Domains chosen by state tobacco control program^a^
**Strategic Planning**			
Access to resources	Well, that’d be really great because [name] was thinking some of these things we might not need immediately for what’s on this action plan, but you’ve got a lot of experience in other states, so it would be really helpful to have something that when we get to that point of development, we’d have something to guide us.	South	Partnerships
	What I’ll do with them [action planning resource] is we’ll do a team meeting, and we’ll do it as a shared document together while we're meeting and get it all done.	Northeast	Communications
**Program Evaluation**			
Meeting CDC Grant Requirements	Our CDC grant, I don’t know if you’re familiar with what the new grant is requiring, but you need to award a community partner, the reaches, kind of some of those priority populations. And so, we did do a lot of investigation into who those populations are. And we’re also, as I mentioned, we’re doing our strategic plan. And so, a lot of those populations are rising to the surface.	West	Partnerships, Communications
	We’re in the process of working on finalizing our strategic plan for tobacco, and our sustainability plan is part of the infrastructure piece of the tobacco strategic plan. And so, we’ll be considering some of these things as part of the plan. I’m going to be sharing the information on the latest evaluation with them as well so we can take that into account.	Midwest	Communications
**Program Adaptation**			
Learning from other state programs	Would you be convening a conference call or a webinar or something with the states that you’ve worked with? Because it’s really helpful to compare notes and talk. I was at a western region policy for western states. And that was really helpful. But those are states that we’re just sort of automatically pulled together because we’re in the West. You’re working with states all around the country.	West	Communications, Funding Stability
	So, I have done a literature review, and I do look at other states to see what they’re doing. I think I downloaded most of them in some form or another, but if you’ve been working with other states and you’re really impressed with the caliber of work that they’re doing, they have innovative ideas, I definitely think it’s a good idea to learn from the best so that we might be able to emulate.	West	Strategic Planning
	Okay, one of the strengths that you guys really brought were those resources from other states. And I know that [other state program staff] are really good at putting together communication plans. . . . If you have a great example of one you want to send, it’s great to just look and learn from others, too.	West	Partnerships, Communications
**Partnerships**			
Partnership building	We’ve improved communications with the Department of Children and Families. They’re the ones who are assigned to sign our compliance assessments. Again, you’d think that we would have a good relationship with them, but we have reached out over the years and they just, in the past, have not been too interested. We have established a better relationship with them in recent months as well. There is definitely some additional partnership building that’s happening.	South	Partnerships, Communications
	So, we developed a work group specifically that met when the network met, to work on just those partnerships and communication pieces, and actually we worked on those outside of our tobacco control network meetings.	South	Partnerships, Communications
	Pulling the partners together for the first time, prior to that, I think we all were accustomed to each other and working together in our own ways, but we just didn’t make time to come together and talk about what our priorities were for sustainability and what our needs were. And to have that meeting and for some of our partners to be able to communicate what their needs were and for us to be able to say, “Well, we have that. We could do that,” you know what I mean? That was really beneficial.	South	Partnerships, Communications

Abbreviation: CDC, Centers for Disease Control and Prevention.

a The Program Sustainability Framework structures programmatic factors into 8 sustainability domains: Environmental Support, Funding Stability, Partnerships, Organizational Capacity, Program Evaluation, Program Adaptation, Communications, and Strategic Planning (2). Each tobacco control program chose to address 1 or 2 domains as part of an action plan.

**Strategic Planning.** In this domain, access to PACT resources, such as the sustainability action planning guide, facilitated program ability to develop sustainable goals. These resources were reported as helpful and a benefit of participating in the study.

**Program Evaluation.** In this domain, states reported that using the Program Sustainability Framework and PSAT facilitated the development of a strategic plan for their grant application to the Centers for Disease Control and Prevention (CDC). This evaluation allowed programs to better plan for future priority populations and needs.

**Program Adaptation. **In this domain, programs expressed the idea that learning from other state programs to understand and potentially emulate their successes would facilitate sustainability. Some programs were evaluating available literature, meeting with other programs at conferences, and using resources from other states. Furthermore, there was strong interest in developing a conference or webinar where people from programs nationwide could meet each other, ask questions, and adapt their programs accordingly afterwards.

**Partnerships.** In this domain, enthusiastic, communicative, and involved partnerships that were built with new and existing concerned groups facilitated program success. This success came from dedicated work toward improving relationships, creating working groups, building partnerships through improved relationships, coming together in workgroups, and strengthening communication about needs.

## Implications for Public Health

This study identified barriers and facilitators to increasing capacity for sustainability among evidence-based TCPs. Organizational Capacity, Environmental Support, Partnerships, Communications, and Funding Stability domains impeded the ability of TCPs to increase their capacity for sustainability.

Within Organizational Capacity, lack of sufficient staff, high rates of turnover, and difficulty hiring new staff made it challenging to cover workload and achieve goals. These barriers came from insufficient resources and resources being directed away from tobacco control work and aligns with prior research documenting that public health systems face critical barriers such as limited organizational capacity and financial resources that decrease their sustainability (9).

The largest barrier to sustainability was lack of legislative support (Environmental Support). Lack of political support has played a key role in inequitable national distribution of public health resources (9). This inequitable distribution ties into Funding Stability barriers, where unstable funding of TCPs prevents sustainability capacity. These domains demand attention because they are most influenced by external factors such as legislative goodwill and state priorities, even though higher levels of public health spending are associated with better population health outcomes (10).

Limitations and impediments of communications was cited as a barrier with partners and the public. Communication is an essential part of effectiveness in public health programs, with consistency, transparency, and frequency being valued by practitioners (11). Programs that create goals or plans that align with these values could improve their sustainability capacity in this domain.

Our study also highlighted facilitators to increasing capacity for sustainability of evidence-based state TCPs within the following domains: Strategic Planning, Program Evaluation, Program Adaptation, and Partnerships. Within Strategic Planning, programs reported that resources from the sustainability workshop and ongoing technical assistance were helpful when planning for the future.

Evaluation of the sustainability of programs, especially when preparing for CDC grant applications, helped TCPs to understand where programs lacked capacity, such as reaching priority populations or infrastructure gaps. Program evaluation is important to the sustainability of public health programs and health programs for long-term effectiveness and improving outcomes (12) and is a valuable way to increase sustainability capacity of public health programs. Furthermore, TCPs made program adaptations as a result of examining data and evidence (13). Therefore, the program adaptation process could be further improved through evaluation and also through collaboration with nationwide networks or working groups.

Uniquely, partnerships experienced facilitators and barriers to increasing program capacity. Success or hindrance in this domain was dependent on factors such as partner enthusiasm, commitment, and relationship strength. Involvement of partners to incorporate evidence-based public health can improve community health improvement plans (14) and sustainability in this domain (15). Throughout public health, common facilitators of partnership sustainability are invested time in relationships, promotion of communication and trust, shared motivations, and reciprocity (15).

### Limitations

Our study has several limitations. First, we included only 11 intervention states. Although programs that participated were diverse in terms of funding, adult smoking rates, and policy progress, they may not be generalizable across all states. Second, we had an unequal number of calls in each state, which resulted from factors such as inadequate availability of TCP program managers, limited participation by some states, and the effects of COVID-19. Third, the duration of the study may have been too brief. Although it was possible to assess changes to sustainability capacity, longer follow-up may have been needed to understand sustainability over time.

### Conclusion

Our study was the first to identify barriers and facilitators to increasing program sustainability capacity of TCPs. Understanding how these factors affect sustainability capacity was accomplished by using the Program Sustainability Framework. Addressing known barriers and enhancing facilitators for programs increases the possibility of achieving program sustainability. Many of the barriers faced could be addressed through methods such as gaining stronger political support, increased funding, and increased staff. Furthermore, cultivating facilitators such as formalized sharing networks and greater technical assistance could assist programs in becoming more sustainable.

Overall, our work advances the understanding of program sustainability capacity and technical assistance for public health programs. Future research should focus on interventions that address ways to overcome barriers to sustainability planning and ways to improve implementation of sustainability planning to better support public health programs.
